# Historical Sketch of the Progress of Medicine in the Year 1807

**Published:** 1808-07

**Authors:** 


### Medical and Phyfical Journal.

VOL. XX.] JULY, 1808. [NO. 113.

*Printed for R. PHILLIPS, by W. Thorne, Red Lion Court, Fleet Street, London*

### Historical Sketch of the Progress of Medicine in the Year 1807; by Mr. Royston.

### No. 2.

( To be continued Annually. )

As the boundaries of science extend, and the stores of literature accumulate, periodical arrangements of the accessions to knowledge become indispensable. There is scarcely a mind so happily constituted, so endowed with the powers of discrimination, as to have a clear and comprehensive view of the entire circle of science, in its present elaborated form. The novelties that daily arise, the changes in nomenclature, the intricacies of detail, or of theory, the plausibility of hypothesis, together, present complicated difficulties, only to be conquered by divi[illegible]ing; a segregation of the different genera of science, ar[illegible] an arrangement of the species of these genera under dis[illegible]ct heads, can alone render perspicuous and easy, a s[illegible]t become perplexed by the extent of its own improven[illegible]

Those who are in the habit, either from inclinati[illegible] duty, of inquiring into the progress of science, wil[illegible] the force of these observations; and will, perhaps, acknowledge some obligation to the pioneers of literature, "who remove rubbish, and clear away obstructions from the paths through which learning and genius press forward to conquest and glory."

In a view of the progress of discovery in the Art of Medicine, Anatomy and Physiology, comprehending a knowledge of the structure, functions, and habits of animal life — Natural History of the Atmosphere, and the effects of its variations on every species of vitality — Chemistry — Botany — Experimental Philosophy, command a priority of attention, as affording the principles, as well as furnishing materials for the rational practice of Physic.

The wild hypotheses, that have from time to time obtruded into Medicine, have led astray less frequently, perhaps, the anatomist, than any other labourer in the field of science. His peculiar business it is to investigate facts by a developement of the structure; and from a knowledge of the structure to deduce the modes of operation in the animal functions. In proportion to the tangible nature of the objects of his investigations, is the precision of his conclusions. Even when he has deviated into the visionary world of muscular motion, his theories have not been mischievous: if they have not convinced, they have not led to destructive practice. If in the earliest ages of the art, the prejudices of mankind compelled him to guess at the internal structure of the human frame by an examination of a link lower in the chain, the refined state of the science now leads him, with increased ardour, to explain more precisely by comparative anatomy,* operations in life, still so obscure, as almost to be thought arcana of the deity. Muscular motion, generation, and digestion, require the decisive elucidation, that marks the bounds between opinion and knowledge. Mr. Home, in an interesting paper read to the Royal Society, has exerted much ingenuity of anatomical research to explain some circumstances in the process of digestion. By an examination of the structure of the stomach in a variety of animals, particularly those which appear to form the principal links in the gradation between the ruminant and truly carnivorous, a considerable advance has been made to explain the process of converting animal and vegetable substances into chyle. The conclusions that are drawn from this elaborate investigation, conjoined with the experiments of Spalanzani and Hunter, support the opinion that the stomach is the only seat of the digestive process; that the gastric juice is secreted by glands immediately attached to the œsophagus: and that the pylorus is chiefly a receptacle for the chyle. Anatomical knowledge has been much advanced by the art of making preparations. Injections of the blood-vessels and lymphatics have materially assisted an inquiry into the structure of these parts; but there is a species

* As the connection of comparative Anatomy with the study of Medicine, is now so far admitted, as to render it an object of importance, we have much satisfaction in recommending Prof. Blumenbach's short Treatise on the subject as translated by Mr. Lawrence. Till the translation of this, a short elementary Treatise on this part of science was wanting in the English language.

species of anatomical imitation, employed by the Italians, and of which we have hitherto seen but few specimens in this country, which, if properly cultivated, might afford considerable facilities to anatomical study. The exquisite perfection of the artists of that country in modelling the different parts of animal structure in *wax*, astonishes by the correct imitation, as much as by the beauty of the workmanship. Mr. Brooks, of the Anatomical Theatre in Blenheim-Street, has models of the organs of sense, executed with inconceivable beauty and precision. They were imported from Florence at a high price, and represent the structure and nerves of the organ of smell, the eye, the ear, and the tongue. Very few, if any attempts have been made in the other countries of Europe, to form these representations of the parts of animal bodies. France, however, is emerging from this negligence; and has established a school, under the superintendance of M. Laumonier, at Rouen, for teaching the art of *anatomical modelling* in wax. Already, it is said, have the specimens at this school, in the exactitude of detail, and in the truth of imitation, emulated those manufactured at Florence. It will not be readily admitted that England cannot furnish artists of skill, in every mechanical operation, equal to any that Italy or France can produce.

The art of Surgery derives all its precision, all its safety, from a knowledge of the structure of the parts it is to act upon: not a step can it advance securely without its guide Anatomy. At no period has this been more fully understood than in the present. The advantages that have arisen from the application of expert anatomists to the scientific and operative parts of surgery, is very evident. The labours of Abernethy, Astley Cooper, Bell, &c. afford strong testimonials of this fact.—In a system of operative Surgery, founded on the basis of Anatomy, Mr. Charles Bell has been very successful in subverting the pernicious and dangerous doctrine, that a minute attention to Anatomy serves but to embarras, and render the surgeon timid and indecisive. The first volume of this work at present only is published. A great number of sketches, taken by the author from actual observation, accompany and elucidate the text. The appearance of the second part of the Anatomy and Surgical Treatment of Crural and Umbilical Hernia, by Mr. Astley Cooper, cannot fail to improve this part of practical surgery. The difficulties attending both the reduction of the Crural Hernia by the taxis, and the operation when it is irreducible, must make a work on this subject, executed as this is, highly acceptable to the profession.* The progress of surgery in the year 1807, has been marked in England, if not by any fact that may be considered as an extraordinary discovery, by a variety of productions of considerable merit, and of great utility in explaining many circumstances in the different branches of this department of the medical art. A Treatise on the Diseases of the Joints, by Mr. Samuel Cooper, the Prize Essay at the College of Surgeons; Critical Reflections on several important practical points relative to Cataract, comprehending an account of a new and successful method of couching particular species of that disease, by the same author; Sir James Earle's Observations on Fractures of the Lower Limbs,—and Remarks on Hæmorrhoidal Excresences, cannot pass unnoticed. Every practitioner who has adopted the method suggested by Mr. Pott, of laying the limb on the side in fractures of the lower extremity, must have repeatedly experienced difficulty in keeping the fractured bones in just approximation. This short Essay, forcibly suggests, especially in fractured thigh bone, the expediency of returning to the old method of laying the patient on the back, and the limb straight. The principle of Mr. Pott, that of putting the muscles into a relaxed state, need not be altogether abandoned. By bending and raising the knee to any point, and securing the limb, if a fractured thigh, by very simple machinery, all the advantages of relaxing the muscles may be had, free of the hazards incurred by lying on the hip. A Treatise on Hernia, by Mr. Lawrence, also a Prize Essay, though it affords no new matter, presents a useful compilation of established facts and authorized opinions. The requisites of sound judgment, assiduous research, perspicuity of style and arrangement,

* The right mode of reducing crural hernia by the taxis is so little understood, that Mr. Cooper's directions for it cannot be too much known. The patient is to be placed in a recumbent posture, the shoulders a little elevated, and the thighs bent at right angles with the body: the knee of the affected side should likewise be carried inward. The surgeon is then to place himself over the body of the patient, and putting both his thumbs on the surface of the tumour, he is to press gently *directly downwards, as if he were endeavouring to press the tumour into the thigh rather than toward the abdomen.* If this pressure is steadily kept up for some minutes till the surface of the tumour is brought even with the line of the crural arch, the hernia may then be pressed toward the abdomen, and will return into the cavity. Much of the difficulty found in returning this species of hernia often depends on the improper direction given to the pressure.

rangement, are evinced in a proportion extremely creditable to the author.

An accurate ascertainment of the state of the Atmosphere at any particular period, or for a series of periods; an exposition of the changes in temperature of climate, arising, perhaps, from causes very obscure; and an illustration of the morbid affections that may be supposed to have been produced, or to have been remedied by such changes, are circumstances that the faculty cannot look on with indifference. Whether certain classes of Disease arise out of some specific property in the Atmosphere itself; whether the air is a mere vehicle for the seeds of epidemics; and if so, what are the particular states of it influencing this property: whether the poisons that it conveys, if it is a mere carrier, are collected from swamps and bogs, loaded with gases from dead animal or vegetable matter, or furnished by effluvias arising from the surface of the living human body, are questions of no small importance. They are questions, indeed, upon which mankind have differed much and often; and upon which opinion is still unsettled to a degree that involves extreme doubt and indecision. The means furnished by modern Chemistry for ascertaining with considerable precision, the component parts of the air; the accurate and extensive registers of the weather, arranged with the prevalence of disease, and a variety of occurrences in nature, particularly in vegetation; and the elucidation that the subject has lately received, afford the means of pursuing the inquiry, and should certainly stimulate to a deeper research. An examination of the change that the climate and temperature of particular places have undergone in a long series of years, with the consequent effects on animal and vegetable life, is a labour, certainly, of no small interest, and probably of some utility. The mode in which the inquiry has usually been conducted, has, however, abstracted a portion of its usefulness. An hypothesis has been assumed, and the subsequent research directed to the collection of materials for its support. Mr. Williams, in Remarks on the Climate of Great Britain, has, perhaps, fallen into this error. In a work abounding with curious remark, and presenting novel and interesting views, the leading principle is, that the humidity and consequent coldness of our modern summers, is to be attributed to an increase of evaporating surface: and in a chapter devoted to the consideration of the influence of a cold and humid climate on the animal œconomy, the author labours to support an opinion that the increasing unhealthiness of Great Britain arises from this supposed change in the seasons. Interesting as inquiries after the change that time and various circumstances have induced in the climate of a country, doubtless are; there is, however, connected with them a degree of incertitude, and a disposition to hypothesis, that lowers their value. An inquiry into the changes produced on Atmospheric Air by the germination of seeds, the vegetation of plants, and the respiration of animals, is a subject of more direct and exact investigation. The alterations, whatever they are, become the immediate object of experiment; and, being in a measure, cognizable by the senses, are consequently more determinate than where recourse must be had to historical records made in remote periods of time, and by persons wanting the means of recording these natural phenomena with correctness. In an elaborate disquisition by Mr. Ellis, it is shewn that in the processes of germination, vegetation, and respiration, the oxygenous portion of the atmosphere disappears, while carbonic acid is formed; and that the carbonic acid produced, always bears a certain proportion to the oxygen gas lost.

Among the trains of morbid derangements supposed to be produced or increased by particular states of the atmosphere, popular opinion, at least, will give the foremost place to pulmonic complaints. The state of the atmosphere throughout Europe, during the summer of 1807, and the degree of disease accompanying this state, but ill accord with the hypothesis just mentioned.

At Paris the heat was intense, with long continued calms; and catarrhal affections, and phthisis pulmonalis were unusually frequent. In London, a longer series of hot weather has seldom occurred; and the frequency of pulmonic complaints in the months of June and July, seemed in some way connected with this increase of temperature. That disorders of the pulmonic system do not, however, always, or even frequently depend on particular states of the atmosphere, is rendered more than probable by Dr. Robert Bree's "Inquiry into Disordered Respiration." The celebrity of this work shews that the faculty have of late entertained more enlarged views of disorders generally referred to the lungs; and that in the investigation of the causes of such complaints, we ought to look farther for their seat. Many supposed pulmonary cases might have been saved, if the reasoning of this author had been earlier applied, and if a treatment calculated to remove local weakness had been advised in the place of customary depletion and general relaxants. The means of removing obstructions from the viscera below the chest, are, in numerous instances, the best remedies for supposed and real pulmonary affection. The whole of this work, a fourth and considerably improved edition of which has appeared this year, offers a new pathology on the complaints called pulmonary, and very important reflections on their relations.

There are classes of disease which possibly arise, in part, from some changes in temperature and climate; but certainly connected with particular forms of society, and habits of life. Nervous complaints, as they are vaguely called, multiply in a ratio according with the approaches to that period of refined society which approximates with mental and coporeal debility. History and uniform experience agree, that the delicate and extenuated beings, whose frames cannot endure the exercises of former times, whose digestive organs nauseate the wholesome viands that supported their ancestors, whose intellects are too nicely attuned to listen, without disgust, even to descriptions of the domestic œconomy of their forefathers, have arrived at an acmé incompatible with undertakings that require strength of mind, and vigor of body. A train of morbid feelings, unknown to earlier ages and the manhood of society, afflict this offspring of luxury and refinement. Hypochondriasis with its proteus symptoms, Hysteria in every varied shape, comprehênd, perhaps, all those fugacious and ill-defined complaints designated by the term Nervous. There can be little doubt, from whatever cause, or causes, it may arise, that the nervous temperature, as it has lately been called, is fast increasing in this country. In a work written expressly with the intent to counteract this predisposition, the causes are pointed out by contrasting the savage state with the last period of refinement. Man, living in all the simplicity of nature, is exempt from those bodily ailments and mental disquietudes, which are produced by the dissipations of civilized life. Health and vigour of body, with passive content of mind, are the inheritance of the untutored savage. In the population of large towns at a period of high civilization, composed of the idle and dissipated; literati; artificers, and manufacturers; those employed in drudgery; persons returned with broken constitutions from the Indies; and females, operated upon by various employments and modes of life, and the vices and preposterous customs of society; we are to look for the origin of physical degradation, and the source of nervous diseases.

diseases. Among the causes that operate in increasing the nervous temperament, those that tend to destroy the spring and elasticity of the mind, are not the least formidable. If literature strengthens the intellect, it is when employed of a proper quality. The passion for novel reading, Dr. Trotter considers as one of the causes of nervous disorders, and reprobates it with just indignation.*

In the military hospitals of Germany, a method of treating Tetanus has been practised with great success. This consists of an alternate use of opium and carbonate of potash. M. Stutz, a physician of Suabia, was led to employ these two substances alternately, by some observations of M. Humboldt in his work on the nerves; and found that one third of the quantity of opium, used in this manner, produced the best effects.

Hydrophobia, as the consequence of the bite of rabid animals, is certainly the most severe and fatal of the morbid derangements in the class *Neuroses.* In the specific disease, every mode of treatment has hitherto been unavailing; death, we believe, has been the uniform termination. An extraordinary degree of alarm was excited in the early part of this year, in the metropolis and its vicinity, with respect to the prevalence of this disease among dogs: many persons were bitten, and excision of the bitten part as the most effectual preventive, was frequently employed: but it is probable few of the dogs were rabid. It is certain, however, that the genuine disease has occurred during the year. Under these circumstances, the attention of the faculty has been drawn to it, in an unusual degree. Dr. Bardsley, of Manchester, has industriously investigated the history and natural phenomena of the canine and spontaneous Hydrophobia. In the progress of the inquiry, he gives an epitome of the most remarkable facts and observations on the subject, dispersed in a multitude of writings; and details at large, the history of a case

---

* "The mind that can amuse itself with the trash of most modern novels, seeks an enjoyment beneath the level of a rational being. It creates for itself an ideal world, on the loose descriptions of romantic love, that leaves passion without any moral guide in the real occurrences of human life.—To the female mind in particular, as being endued with finer feelings, this species of literary poison has often been fatal. How cautious, then, ought parents to be in guarding against the introduction of these romances among their children; so calculated to produce the morbid sensibility which is to be the bane of future happiness. It is lamentable that three fourths of these productions come from the pens of women; some of whom are known to have drank deep of the fountains of pleasure and adversity."

case of Hydrophobia occuring twelve years after the bite of a supposed mad dog. An interesting part of the investigation relates to the origin of canine madness, and the means of its extirpation. From a full view of the subject, he concludes.

1st. That the disease always originates in the canine species.

2nd. That it never arises in them spontaneously.

3rd. That the contagion, when received by them, never remains latent more than a few months.

On these plain facts he founds his plan of extirpation. It consists merely in establishing an universal quarantine for dogs within the kingdom, and a total prohibition of the importation of these animals, during the existence of such quarantine.* Though every endeavour to establish a successful mode of treating canine Hydrophobia has hitherto proved ineffectual, yet all hope of future success should not be abandoned. Perhaps the use of opium with carbonate of potash may deserve attention in this as well as in Tetanus. A decoction of the *Celtis Ambralis* † is said to

---

* With a conviction that the faculty will give every assistance to farther the patriotic views of Dr. Bardsley, we endeavour to give additional publicity to those views, by inserting the following queries, to which the author requests answers, addressed to him at Manchester.

1st. Is it not highly probable that Canine Madness in dogs arises solely from the actual communication of the virus?

2nd. Is not a few months the longest period, during which the poison lurks dormant in these animals?

3rd. Does it not appear sufficiently probable, that a proper system of quarantine, extending to dogs within the kingdom, and to such as may be imported, would be the most effectual means of extirpating Canine Madness from the British Isles?

† Probably the *Celtis Australis* is meant, which grows abundantly in the South of Europe, and is sometimes used in Dysentery and Menorrhagia. The extent of the vegetable kingdom makes it often difficult to identify particular species of plants with a correctness sufficient to employ them medicinally, with some prospect of success. This difficulty is certainly felt with regard to the *Celtis Ambralis*. In the last edition of the *Systema Naturæ*, there are six species of *Celtis* enumerated; the *C. occidentalis*, a native of Virginia. (Mill. fig. tab. 88.)—*C. orientalis*, Levant. (Pluk. alm. t. 221. Rheed. Mal. 4 t 40.)—*C. Americana*, West Indies. (Plum. Cat. 18.)—*C. aculeata*, Caribbees (Swartz prodr. 53. obs. 92.)—*C. lima*, West Indies (Swartz prodr. 53.—*C. micrantha*, Jamaica, (Brown Jam. 173. tab. 12, fig. 2). If any of the species of Celtis are indigenous to Spain, it is probably the *Orientalis*), but that is a deciduous forest tree of large growth, not an herbaceous plant, as the account indicates. The *Phytolacca decandra*, in the form of the powdered plant, is said to have cured a spasmodic affection following the bite of a rabid dog. The *Phytolacca* is used as a common purge in N. America, and some empirical attempts have been made with it to cure Cancer. It certainly possesses active qualities.

to have been employed by the shepherds of Andalusia with great success in Hydrophobia since the year 1801; and Mr. Blane has made public a recipe for curing the bite of a mad dog, as used upwards of 150 years at Tring in Hertfordshire, with extraordinary success. Every county in England has some individual who possesses a secret for curing this dreadful disorder; a circumstance that diminishes any confidence that might be given to such nostrums. Still, however, society is indebted to Mr. Blaine for procuring and making public one of them, as affording the means of ascertaining its properties. This medicine, a recipe of which is given below,* is stated by Mr. Blaine to have been very uniformly successful in the prevention of Hydrophobia; and that it produces nausea, giddiness, tremors, and cold sweats; these effects last two, three, or four hours, and leave the person free from all ailment.

The propagation of an Ophthalmia by contagion, originally imported from Egypt, has been farther confirmed by Dr. Vetch; who maintains that it is communicated by no other means than a direct contact of the morbid discharge. This circumstance forcibly marks the propriety of separating the diseased from the healthy. In the army, in manufactories, and in schools, this precaution should be religiously observed. Though the various diseases of the eye have been familiar to British practitioners from the time of Bannister, who enumerated one hundred and thirteen forms of morbid affections of this organ; yet the Egyptian

---

* Copy of an original recipe, which has been in the family of John Webb for 150 years.

Take of the leaf and tender bud of rue half a large tea cupful, when cut quite small, the cup to hold about a quarter of a pint, beer measure; take the same quantity of large or tree box, also cut this small; add nine leaves of red sage, cut small; let them be without blemish. Take half a pint of new wheat-meal from the mill, or good fine flower, and about one table spoonful of yeast; mix it together as dough; let it lay about half an hour, then bake or boil it; take one third of it in new milk each morning. This quantity for a man or woman, sheep, hog, or dog: but for a cow, or horse, take one large tea cupful of rue, cut small, and the same of box, but only nine leaves of sage; give this in milk or some other liquid. Half this quantity will do of the rue and box for a colt or calf; but nine leaves of sage. My father, (says James Webb,) has cured some men when mad: then he took one tea cupful of the rue and one of the box, and nine leaves of sage; boil it well in a pint of milk, and give it as quick as possible. Half this quantity of rue and box for a small child; but in all cases neither more nor less than nine leaves of sage.

The rude structure of this recipe proves its authenticity, independent of its being given on oath, by James Webb, the son of John Webb.

Egyptian Ophthalmia presented phenomena either so new, or aggravated to a degree of violence before unknown, that much diversity of opinion existed as to the most effectual modes of treatment. Cold and heat, sedatives and stimulants, in conjunction with topical and general evacuations, were employed, commonly without arresting the violence of the disease; or, only retarding its destructive termination. Under these circumstances it was determined to push the mode by depletion to a maximum, that should, at least, decide very fully, its powers over this local inflammation. The event was fortunate. A use of the lancet, free beyond precedent in modern times, was found to keep in subjection this formidable, and under milder treatment, this unmanageable disease. The abstraction of thirty, forty, fifty, or even sixty ounces of blood from the system, was found to suspend the morbid action, and prevent the disease arriving at its second stage.

If enthusiasm is to be indulged in any branch of science, perhaps it is in natural history, and in the pursuit of Botanical Inquiries. What has led the philosopher of Europe to traverse the wilds of Africa, to explore the forests of the American continent, to contend with the stormy seas that surround the capes, or to brave the eternal frosts of the polar circles; to investigate nature in her remote and inhospitable climes, where peril in every form attends on his progress, but that enthusiasm of soul which impels to the endurance of privation, and to the encounter of danger? To Linnæus, Park, Vaillant, Cook, Bruce, is science indebted for facts which support its principles, adorn its details; give interest to its investigations, and conviction to its doubts. If the traveller, who pursues his discoveries, frequently at the hazard of life, deserves a distinguished place in the temple of science; no dishonourable station among those who have laboured for the advancement of knowledge, will be allotted to the patient scholar, who in his closet arranges the materials, and moulds into system the fugitive facts that have been collected in the wilderness, on the bosom of the ocean, in the recesses of the mine, on the summit of Mont Blanc, or the stupendous heights of the Cordilleras*.

Among those who have deserved the thanks of society for this labour of arrangement, this generalization of facts, the

* In this vast mountainous chain, which stretches from Carthagena to the straits of Magellhan, there are some points of immense height. Chimboraço is 19,320 feet above the sea, and El-Corason, the highest which has been yet ascended in America, is 15,220.

the president of the Linnæan society holds a distinguished place. But chiefly are we indebted to him for his philosophical views of Botanical Science, and particularly in 1807, for an introduction to philosophical and systematical Botany; an elementary work in which the first principles of this delightful branch of natural knowledge are divested of their forbidding aspect, made to interest the feelings, and awaken the curiosity. Those who have felt the consolation that Botany affords in the retirement of the country, and in the solitary stations often appointed to genius, will acknowledge how much they are indebted to the talents of Dr. Smith, for the elucidation of a science once, but now no longer difficult. To the student in medicine, and even to those who think the days of study passed by, this introduction cannot be unacceptable. The ignorance of physicians and apothecaries in Botany, is enumerated among the obstacles to the improvement of the medical art. Ignorance, at least of the indigenous plants, of their classification in systems, of their medicinal properties, or of their poisonous effects, is unpardonable in those who profess to have studied and to practice the healing art. The study of Botany seems peculiarly appropriate to the practitioners in medicine who reside in the country, and who should reflect that there are many plants in the British Islands, whose powers remain unexplored; and that it may be the lot of some fortunate individual to discover the virtues of unnoticed species, and, perhaps, to develope some new property in the vegetable kingdom. But few years have elapsed since Digitalis was no otherwise known than as one, and perhaps the least efficient ingredient, in an ointment for strumous ulcers. An accident led Withering to an investigation that has brought into use a remedy of peculiar and of astonishing powers*. The subsequent labours of Darwin, Ferriar, Fowler, &c. have taught the practitioners of medicine to know in it, the most powerful moderator of the action of the heart and sanguiferous system.

Dr. William Hamilton, availing himself of the opinions and discoveries of preceding writers on this plant, to which he

* This medicine may almost be said to be possessed of a charm for allaying the inordinate action of the heart and arteries; and in this point of view, as well as for its efficacy in some kinds of dropsy, particularly hydrothorax, its introduction into medicine is one of the greatest benefits our science has received in modern times. Currie's Medical Reports, Vol. ii. p. 419.

he has conjoined no inconsiderable experience of his own, has given a view of its medical history, and its cultivation; of its utility in hydrothorax, and other effusions of water into the cavities of the chest; in phthisis pulmonalis, hæmorrhage, scarlatina, and measles. The investigation of its powers over these diseases, is preceded by a review of whatever has been written upon it for the last thirty years.

The action of Digitalis upon the systems of some animals* is very manifest: yet the laws by which its operations are governed, are so obscure in some, if not in many instances, that an inquiry into its properties and its effects, still excites peculiar interest. We are taught that when used internally, it diminishes the irritability of the system, it decreases the frequency of the pulse, and increases the action of the absorbents. Withering, however, observed, that with persons of tense fibre, of great natural strength, labouring under ascites or anasarca, the Digitalis seldom succeeded; and that, on the contrary, when the pulse was found feeble or intermitting, the anasarcous limbs and body soft and yielding, the countenance pale, and the skin cold, the diuretic powers of the plant were conspicuous. These observations, as far as they concern ascites and anasarca, are confirmed by Dr. Hamilton. In the hydrothorax, however, he found the Digitalis succeed in the opposite state of the system.†

It is also asserted, that in pneumonic inflammation Digitalis cannot be depended on till the tone of the system has been much reduced by previous blood-letting. The same difficulty obtains in phthisis, in proportion to the inflammatory disposition of the vessels.‡ This apparent contradiction

---

* Rabbits feed on the green leaves of the digitalis with impunity.

† Dr. Hosack, Professor of Botany in Collumbia College, says, In the 1st. or inflammatory stage of phthisis P: digitalis was useful in removing pain and soreness of the chest, in lessening the frequency of the cough, in facilitating expectoration, and in diminishing febrile action in general; but in the advanced stage, it sunk the strength of the patient, destroyed the appetite, and in all respects increased the violence of the complaint. In one case of incipient phthisis, preceded by hæmorrhage from the lungs, it operated like a charm in relieving the patient from some of the most formidable symptoms of the *inflammatory stage.* Digitalis will also probably be found a valuable medicine in the treatment of most inflammatory complaints, and may be no less useful in pleurisy, peripneumony, or inflammation of the brain, than in the *first stage* of phthisis. *De Lile's Inaugural Dissertation.*

‡ Dr. Sanders, in a recent publication on pulmonary consumption, contends that digitalis *is a powerful stimulant, that it increases the strength and*

diction requires further illustration. If Digitalis diminishes the frequency of the pulse, and lowers the irritability of the system, it might be supposed by those who are not in the habit of pushing refinement to the verge of obscurity, that it would, at least, be a useful auxiliary to bleeding, and other evacuants. Yet is it urged, that the use of this vegetable, the most remarkable property of which is its power of diminishing the action of the heart, is inadmissible, until the diseased action connected with the inflammatory diathesis is first reduced by bleeding. So much at variance are opinions with respect to the action of Digitalis, as well as of the proper state of the system for its successful exhibition, that much remains yet to be done for a full and clear developement of its powers. So certain, however, is it that digitalis is not one of those inert substances upon which investigation is thrown away, that the faculty are called upon to ascertain positively its properties, and to discriminate the diseases and the states of the system in which it may be useful, with a determined precision that will set this contrariety of opinion at rest.*

The utility of botanical studies is not confined to the *materia medica.* The elucidation of properties in plants applying to domestic œconomy, is not altogether foreign to the views of the medical practitioner: and it is his peculiar province to detect the poisonous principle that exists in many vegetables; to point out the appearances that indicate this virulence, to describe the symptoms that arise from its application, and to supply the means of cure. The detection of poisonous plants, has been thought an object of such importance by the Imperial Academy of sciences

---

*and frequency of the pulse, and if continued sufficiently long, produces flushed face, head ache, hot skin, restlessness, and all the symptoms of febrile action.*

* A fact stated by Dr. Baildon in the Edin. Med. Journal, July, 1807, may assist in explaining from whence has arisen the diversity of opinion on the action of the digitalis. This gentlemen observed in his own case, and repeated the experiment a great many times, that after the digitalis had taken effect, *his pulse was not lessened in frequency when he stood erect;* it was then upwards of a 100. When he sat down, it fell considerably; when lying on his back, it fell much more. Thus, during the time it was at 40 when lying, it was about 75 when sitting, and above 100 when standing. Dr. Baildon found the pulse to vary in this manner in all the patients to whom he gave the medicine in any extent. This is a fact of the most interesting practical nature in the medical history of digitalis; and calls for that minute attention on the part of the faculty, that shall either establish it as a principle in the exhibition of this powerful remedy, or reject it as one of those accidental effects that arise from inexplicable causes.

sciences at St. Petersburg, that it has offered a prize of one hundred Dutch ducats for the best memoir describing the easiest and most certain method, by which any individual not having a knowledge of Botany, may detect venomous plants, in a short time, at a small expense, and in a manner perfectly decisive.

The general opinion that deleterious plants have a lurid and poisonous aspect, denoting their qualities, though sometimes true, is subject to many exceptions. The *Prunus Lauro cerasus, Lin:* remarkable for the beauty of its leaves and the elegance of its flowers, is poisonous; the numerous and beautiful genera of ranunculus and anemone, are for the greater part noxious. The black, shining fruit of the *Atropa belladonna* has been frequently fatal; and the roots of the *Conium maculatum* have been eaten for parsnip, and have occasioned singular derangements of the nervous energy, sometimes ending in death. The *Oenanthe crocata,* perhaps the most active of the vegetable poisons, has nothing in its appearance that forbids its being mistaken for some semi-esculent plant. There are many instances of its having thus been fatal. As there are many plants of inviting exterior possessing noxious qualities, there are also several whose appearances indicate, according to the common notion, venomous properties, which in reality, are not merely innocent but useful. The *Arctium lappa,* the family of *Chenopodiums,* the *Solanum tuberosum,* and *Capsicum,* have this deleterious exterior. The *Sison inundatum,* the *Phellandrium aquaticum,* and other umbellate plants growing in marshes, have been stigmatized, perhaps unreasonably, as poisonous. The *Sison inundatum* is, however, objectionable from the *Oenanthe crocata* having been taken for it: and the *Phellandrium aquaticum* is at least doubtful. It is fatal to horses; but this possibly arises from the *Curculio parapleticus,* which inhabits its stem, being eaten with it. The criteria distinguishing poisonous agarics, are yet indeterminate: and the repugnance of animals to pernicious plants, is under a similar ambiguity. The avidity, and the impunity with which rabbits devour the *Digitalis purpurea,* noticed in a preceding page, affords a striking instance. The pleasant or the disagreeable odour of plants is no less fallible. The pernicious, and sometimes fatal, effects of the perfume of the lilly is well known; and the odour emitted by the *Crocus sativus,* though not in the least disagreeable, is found under certain circumstances of accumulation, to occasion serious soporific effects. M. Sage, a member of the French

National

National Institute, describes these effects, as occuring in the *Gatanais*, one of the former provinces of France, where this plant is cultivated in great abundance. "The farmers after collecting the flowers of the saffron in the autumn, carry them home and spread them on linen sheets in their houses. In the evening the females are employed in picking off the pistils, the odour from which produces the most alarming effects on the nervous system. The disease thus induced is vernacularly termed the sleepy fever ; it never appears but during the saffron harvest, which usually lasts a month. The narcotic effect of this odourous emanation greatly resembles that produced by opium, and in feeble persons and children it is capable of occasioning death. Like the affection induced by opium, it is best remedied by the employment of vinegar." In some cases, where the torpor has been so great as to resemble death, recovery has been effected by friction, and the application of flannels wet with the acetous acid.

The National Institute of France has this year been much employed on botanical investigations. M. de Beauvais, * M. de Candolle, and M. du Petit-Thouars, have signalized themselves on this subject. The first of these gentlemen has particularly attended to the fructification of a class of plants, whose organs are so minute and concealed, that Linnæus arranged them under a class to which he gave the term *Cryptogamiæ*. M. Beauvais has been so successful in his investigations of this obscure subject, that he thinks himself entitled to give to the Linnæan class *Cryptogamiæ*, the new term *Œtheogamiæ*. Messrs. de Candolle and du Petit-Thouars have attended particularly to the physiology of plants, and to the *Materia Medica*. In memoirs, presented to the Institute, they have investigated the structure, and explained the functions of plants ; and have added to the catalogue many new genera. M. du Petit-Thouars has began to publish a Flora of the island of Madagascar ; and M. de Candolle, beside distinguishing the various plants confounded under the name Ipecacuhana, has made considerable researches into the agreement in the virtues of plants of the same natural family ; and has explained, according to new views,

* M. Palisot de Beauvais now occupies the place in the botanical class of the French Institute, formerly filled by M. Adanson. M. de Beauvais is as well known by his travels in Africa and America, and the floras which have been their result, as by his researches on cryptogamous plants.

views, the rules to be followed in these inquiries.* Every country which has at all attended to the science of medicine, has been sensible of the great advantages resulting from some principle pointing out the similarity of properties, that reside in the individuals of every natural family.

At Ratisbon, the first number of a collection of poisonous plants engraved on stone, has appeared. The prints resemble, and are equal to, well executed wood cuts. This number contains ten prints, with the generic and specific characters annexed. The Napellus acon: the Anemone pratens: Caltha palust: Delphinium staphysag: Helleborus fœtid: Helleborus nig: Ranunculus flam. Ranunculus ficar: Ranunculus acris, Ranunculus scelerat: are delineated with sufficient accuracy. The author of this work, Mr. Keyser, has attached a chapter, explaining the methods of discovering an accumulation of mephitic gas in any situation, and pointing out the best means of dissipating it with safety. Among the literati of Germany, who are constantly employed on objects of laborious research, of wild speculation†, or of heated imagination; it is a circumstance of great gratification to observe a person of the highest rank in society, engaged in the service of science. The Arch Duke John of Austria, is at this time employed on a grand botanical work, in which is given a description of a great number of plants hitherto unknown; of which specimens have been found in the Tyrol, in the county of Saltzburg, and in Lower Austria. Some sheets of this work, ornamented with a great number of plates, have appeared; but booksellers are not permitted to sell them: the Royal Author destines the whole impression for his particular friends, and persons distinguished in science.

The spirit of botanical investigation and discovery has spread from the southern parts of Europe to the extre-

---

* M. Decandolles *Essai sur les Propriétés Medicales des Plantes, comparées avec leurs Formes exterieures et leur Classification Naturelle,* 4to. *Paris,* 1806, we recommend as an ingenious exposition of a subject which has excited the attention of Camerarius, Linnæus, Jussieu, Vogel, Gmelin, Platz, &c. &c.

† The wild notions of Gall, of which we gave last year a short analysis, have fallen into that neglect and disrepute, which we then predicted must happen. The account of his theory of physiognomy, as founded upon the anatomy and physiology of the brain, and form of the cranium, with critical strictures, by the celebrated Hufeland, has been translated from the German into English this year; and has had a direct tendency, notwithstanding the bias of its ingenious author, to overthrow the system, by making it more known.

mities of the north; at the same time that the Spanish government is encouraging the study of botany in its dominions in South America, the Emperor of Russia is extending the knowledge of this science, by sending *M. Redowski* on an extensive botanical tour to the most remote north east districts of Asia, including the islands between Japan to the southward, and the coast of North America to the eastward. The botanists who are occupied in the completion of the Flora of Peru, have presented to the Spanish government, eleven drawings, perfectly executed and coloured from nature, on the spot, of so many new species of Quinquina, which were sent to Spain from Peru in the month of January this year, by Don Juan Tafalla, and Don Juan Mazanilla, both eminent naturalists. If the above are in reality new species, and not simply varieties, we have now drawings and descriptions of twenty-nine species of Quinquina. Thirty additional new species are still expected to arrive; and which, it is understood, have been long known to exist in Peru. In the United States of America it is not surprizing that a spirit of botanical inquiry, and a lively application to natural history, should exist in a very marked degree.* The boundless Flora of that country, the undescribed forms of animal life that probably exist in its almost interminable forests, excite to exertions which the naturalist of Europe is not called upon to make, but when he visits these remote and uncultivated regions. The hope of seeing and describing something that has not before been seen or described, is a feeling common to all mankind. On this immense continent this laudable desire may be gratified to the point of satiety. If the unwieldy Mammoth † is no longer to be found in a state of animated existence, the chasm which his

* Dr. David Hosack has established a botanic garden at Elgin in the vicinity of New York, upon an extensive scale, and which from its local advantages, promises to add very extensively to the present stock of botanical knowledge. From this garden, and from the exertions of Dr. Hosack, who is Professor of Botany in Columbia College, an extensive exposition of the Flora of the American continent may be reasonably expected. It was established in 1801, and from the catalogue published last year, it appears that it already contains 2000 species. A garden similar to the above is about to be established near Boston, and connected with the University of Cambridge; another at Charleston, South Carolina; and a third in New Jersey.

† A complete Mammoth has been found in a state of perfect preservation, on the borders of the frozen ocean. It was discovered by Schoumachoff, a Tungoose Chief, in the autumn of 1799, in the midst of a rock of ice.

his loss has occasioned in the chain of life, is probably supplied by creatures as wonderful in their peculiar natures. Thousands of non-descript plants may be reasonably expected to exist in wilds where the foot of man has not yet trod. The travels of Mr. Ash into the countries bordering on the Mississippi, and which are in a state of preparation for the press, may be expected to supply many new facts and discoveries, both in natural history and antiquities. The course of these exploratory travels lies along the western part of the Allegany mountains to the Mexican gulph, down the Monongahela, Ohio, Mississippi, and other great rivers; and through the Ohio, Virginia, Kentucky, Indiana, and upper and lower Louisiana States.

The interest excited by accounts of remote regions has a very extensive influence: the novelty of customs and manners, the general appearance of countries, and the productions of various climates, are circumstances, however, of different degrees of importance. The principal advantage, perhaps, arising from the travels of individuals, or from the expeditions of discovery in which governments have of late years engaged, is the transfer of the natural or artificial excellencies of one country to another. By this means, the nations of Europe have made the productions of India, of America, of Africa, and the numerous islands of the South Sea their own; and have returned the arts, manufactures, and science of polished life. The catalogue of native British plants, either useful or ornamental, is probably very small. Fruits of every kind, esculent plants, the *Solanum Tuberosum,* and even the varieties of *Triticum,* were foreigners. They are now, however, become denizens; and we are indebted to the President of the Royal Society for observations that may lead to a naturalization of many others. The possibility of effecting this object is clearly proved by the fact of many plants which were formerly kept in stoves, being now raised in the open garden. In annuals this change is effected with little trouble. All that is required is to enable them to ripen their fruit in a comparatively cold summer; after which the hardest frost will have no power to injure the seed. It is more difficult to inure the perennial: for it has to encounter frosts with its buds and annual shoots, that have sometimes been so severe as to rend asunder the trunks of our indigenous forest trees. In 1791, seed of the *Zizania Aquatica* was procured from Canada, and sown in a pond at Spring Grove, near Hounslow. It grew and produced strong plants, which ripened their seed; this seed vegetated in the succeeding spring, but the plants produced were weak, slender, not half so tall as those of the first generation, and grew in the shallowest water only. The seed of these plants produced others the next year sensibly stronger than their parents of the second year. In this manner the plants proceeded, springing up every year from the seed of the preceding one, becoming annually visibly stronger aud larger, and rising from deeper parts of the pond, till 1804, when several of the plants were six feet high, and the whole pond was completely covered with them. This experiment proves, that an annual plant, scarcely able at first to endure the ungenial summer of England, has in fourteen generations become as strong and vigorous as our indigenous plants, and as perfect as in its native climate. From the facts and observations contained in Sir Joseph Banks's ingenious inquiry, it appears that the most certain way of naturalizing foreign plants, is to sow the seeds of such as occasionally ripen them in our climate, after the example of the experiment with the *Zizania**. Very lately a quantity of the seed of the *Chenopodium Anthelminticum* has been sent from America to Dr. G. Pearson, and will afford an opportunity of attempting, on the principle suggested by Sir Joseph Banks, to naturalize a plant, which is said to be a certain and safe remedy for the human intestinal worm † As there is some doubt of the true species of this *Chenopodium*, it is still more desirable to cultivate it. ‡

The sophistication of parcels of drugs, with substances either inert or deleterious, is to be lamented when accidental,

* In the Transactions of the Royal Irish Academy there is a paper on this subject by Mr. Templeton, which has since been inserted in the 2d vol. of the Repertory of Arts.

† A newly observed genus of intestinal worm has been described by M. Laennec of Paris, to which he gives the name of *Distomus Intersectus*.

‡ The *Semen Santonicum* of the shops, is said by Saunders, to be the produce of the *C. Anthelminticum*; and this opinion is adopted by Murray, (*Apparat. Medicam. tom. iv.* § 456) who gives a botanical description of the plant. But doubt still exists on this subject; and the circumstance of the seed sent to Dr. Pearson producing the *Chenopodium Ambrosioides*, in no degree lessens the difficulty. Though we are not yet in possession, at least from this source, of the *C. Anthelminticum*, our disappointment is decreased by the production of a plant which Plenck and Mick used with success in the cure of *Chorea*. In Chalmers, (*Diseases of South Carolina*, p. 71.) in Barton, (*Collections towards a Mat. Med. of the United States, part I. pp.* 38—60.) and in Mease, (*Philadelph. Med. Mus. vol.* 2.) additional particulars of the C. Anthel. are found.

tal, but when it arises from a nefarious intention it deserves to be reprobated in the severest manner. In the city of Hamburgh, a kind of Angustura Bark has lately been sold, that operated as a poison. In some instances in this country, a bark sold for the Angustura, occasioned an approach to syncope, accompanied with tremor and spasmodic twitchings: in other instances it produced the most active spasms. The true Angustura Bark, when good, *is distinguished by having the outer surface more or less wrinkled, and covered with a coat of greyish white, below which it is brown, with a yellow cast: the inner surface is of a dull brownish yellow. It breaks short and resinous. The smell is singular and unpleasant, somewhat like dried fish, (especially when kept close in large quantities) but not powerful. The taste intensely bitter, and slightly aromatic; in some degree resembling bitter almonds, but very lasting, and leaving a sense of heat and pungency in the throat. In powder it is not unlike Indian rhubarb. It burns freely but without any particular smell. In infusion, two drachms to two ounces of boiling water, it does not form a black precipitate with a solution of sulphate of iron, and gives a yellow die to linen.*

*The spurious sort differs from this in having a thicker and more pithy external coat, generally whiter, with spots of a ferruginous appearance; beneath which it is of a light brown or ash colour, often verging to grey: it has no peculiar smell, but the taste is intensely and nauseously bitter, remaining a long time about the root of the tongue. The infusion immediately forms a black precipitate, and merely gives a slight tinge to linen.* There is no doubt but this spurious sort, sold under the denomination of East India Angustura, is really the bark of the *Nux Vomica tree;* and a strong decoction of it given to animals, has occasioned convulsions that in ten minutes have terminated in death.

The Report of the Royal College of Physicians of London, on the subject of Vaccination, affords the most prominent feature in the history of this discovery during the year 1807. On this Report various opinions have arisen. It appears, however, on the face of it, to be composed with a serious regard to truth and candour, and with a reverence for science, in these flippant times, singularly commendable. In its detail, it is clear, manly, and judicious, producing every fact for Doctor Jenner's discovery that a dispassionate inquiry can warrant; and facilitating, on the most rational grounds, a remuneration from a grateful country to a distinguished benefactor of the human race. Concise and perspicuous, compressed but free

 from from obscurity, there are in it particular positions, which cannot be too often repeated.

It is stated in this Report, that Vaccination is, in general, perfectly safe; the disease excited by it is slight, and seldom prevents those under it from following their ordinary occupations. It has been communicated with safety to pregnant women, to children during dentition, and in their earliest infancy. The security derived from vaccination against the small-pox, if not absolutely perfect, is as nearly so as can, perhaps, be expected from any human discovery; for among many thousand cases, with the result of which the College have been made acquainted, the number of alleged failures have been surprisingly small, and form no reasonable objection to the general adoption of the practice. The testimonies before the College of Physicians are decisive that Vaccination does less mischief to the constitution, and less frequently gives rise to other diseases than the Small-pox, either casual or inoculated. The College states this the more strongly, because it has been objected to Vaccination that it produces new, unheard of, and monstrous diseases. Of such assertions, no proofs have been produced; and after diligent inquiry, the College believe them to have been either the inventions of designing, or the mistakes of ignorant men. In its mildness, its safety, and in its consequences, the individual will find the peculiar advantages of Vaccination. The benefits which flow from it to Society, are infinitely more considerable; it spreads no infection, and can be communicated only by inoculation; in all respects, it possesses material advantages over inoculation for the Small-pox. The most forcible argument against inoculation for the small-pox, arose out of the actual increase of deaths from the casual disease, since its introduction. From this objection Vaccination is completely and absolutely free.

These are conclusions from a body of evidence, taken by persons most competent to judge of its credibility as to the fact; and from education and habit, most decidedly master of the subject, as physiologists and practical physicians. When a corporate institution, placed in the most conspicuous station of science, who has to answer to the world for its opinions, and who is certainly neither the partizan of the speculative philosopher, nor the trading projector, pronounce in favour of a discovery, there are few unprejudiced persons who will not admit the force of its decision.

It

It has now become desirable to all nations, to ascertain with the utmost precision the natural phenomena of a morbid affection, so mild in itself as the vaccine disease, and yet possessing the astonishing property of freeing the constitution of its susceptibility for the small-pox. It is scarcely, however, a secondary object in this inquiry, to discover and promulgate the most certain and effectual mode of preserving the vaccine virus in an active state, for a length of time, and during a removal to distant regions. The promoters of vaccination have been laudably employed on this subject, and various have been the projected methods. Among these, a proposal made in 1802, by Mr. Bryce, to preserve the dried scab of the vaccine pustule, and which he asserted, afforded when moistened with water, an active fluid after a lapse of several weeks, has become interesting from the Report of Mr. Shoolbred on the state and progress of vaccination in Bengal.

The extreme difficulty of keeping up the disease in a very high temperature of the atmosphere, induced Mr. Shoolbred to try a variety of methods for the preservation of the virus. The armed lancet, the impregnated thread, the plain glass plates, the glass plates with a cavity for containing the matter, the thorn, the ivory lancet, and the exhausted tube of M. Giraud, were all tried with various degrees of success; but all of them were found precarious, except Giraud's invention. In these tubes the virus continued fluid as when taken from the pustule for eighteen days; and was believed to retain its infecting quality for a much longer time. But a more simple and perfectly effectual method was resorted to. It was found, that the peculiar scab or crust, into which the vaccine vesicle is gradually converted toward the conclusion of the disease, contains the virus in a dry state, with unimpaired activity, for a great length of time. From one month to fourteen weeks, Mr. Shoolbred ascertained that its activity continued. A piece of this scab bruised on glass or any hard body, with the point of a penknife, in a small drop of cold water, so as to produce a turbid fluid, is the simple method of preparing the dried virus for use. This turbid fluid inserted into the arm, in the usual mode of infecting, is effectual in producing the genuine disease.

Mr. Shoolbred observes, "from the prohibitory cautions of Dr. Jenner, and some other writers, not to take the matter for inoculation after the formation of the *areola*, it might naturally be expected, that, in using the scab at a still later period, there would be some risk of producing what they call a spurious disease." But this author, from extensive experience, is satisfied that there is no ground whatever for such apprehension, as he has never seen more perfect vesicles, nor a disease more regular and compleat, in every respect, than that which has been uniformly produced by the scab. This is an inquiry, not of idle speculation or mere curiosity. It is of the first consequence to know, if the disease can, with certainty, be thus propagated; and with positive efficacy as a security against the Small-Pox.

An apparent deviation in Mr. Shoolbred's practice from the principles laid down by Dr. Jenner, for a successful vaccination, renders an inquiry into this subject highly interesting. If it were hazardous to inoculate with virus taken after the areola is formed, how does it happen that Mr. Shoolbred has been so successful with virus, as it seems, of a much more advanced period? Perhaps a more precise knowledge of the structure of the vaccine vesicle may explain this. We have been instructed, that a successful vaccination can only take place when the matter is taken on or before the 9th day; that it should be perfectly transparent; that the punctures should be as superficial as possible, and not more than one made in each arm; and that any excess of inflammation should be immediately repressed by cold and astringent applications. Dr. Adams, whose researches on morbid poisons are interesting equally to the philosopher and the physician, has observed, that it is a most important consideration carefully to avoid using vaccine virus in the smallest degree *turbid*. The character of the true vaccine fluid is a transparency so perfect, that on fine glass, or on the polished point of a lancet, it cannot always be perceived. *To its crystalline transparency* (he says) *there is no exception.* And yet in Mr. Shoolbred's practice, which was very extensive, a *turbid* fluid, procured by rubbing the scab with water, was effective in producing the genuine disease. Notwithstanding this apparent contrariety in a fact or law in the vaccine disease, not much doubt exists but that a due investigation will reconcile the method used in India with the principles laid down by Dr. Jenner*.

Philanthropy

* The mode of inserting the virus in the arm, and the progress and termination of the vaccine vesicle, are so minutely and so correctly described by

Philanthropy will rejoice to find a discovery promising such incalculable blessings to the human race, supported in the British dominions by the decisive Reports of the Royal Colleges of Physicians of London, of Dublin, and Edinburgh. In other parts of the world, vaccination has met with an ardent, if not a similar support. M. Champagny has presented to the Emperor of France, a report of the present state of vaccination in that country. He had received an order from the government to obtain authentic materials for this report, by inviting the prefects and clergy of all the departments in the empire to transmit to Paris official documents on the subject. It appears from this report that vaccination has met with some opposition from the ignorant in France, in consequence of the translation of anti-vaccinarian pamphlets from the English language. By a royal ordonnance, the King of Bavaria has decreed that every child within his dominions, under three years of age, shall be vaccinated before the 1st of July 1808; and that every infant born in future in his dominions shall undergo this inoculation within three months after its birth. Heavy penalties are inflicted on neglect of this decree, and the inoculation of the small pox is prohibited under pain of imprisonment. Instructions for vaccination, accompanied with engravings, drawn up by the vaccine society of Copenhagen, have been translated into the Icelandic language, by M. Thorarsen. Dr. Anderson, physician general and president of the Medical Board of Madras, has given some interesting facts on vaccination. From the statements of this gentleman it appears, that no serious alarm has been caused by the small-pox in that vast extent of country, now subject to Great Britain, in India, since the introduction of vaccine inoculation; nor has the vaccine matter, though transferred from one human subject to another for four years, produced any other disease. Under the direction of the Presidency of Madras, vaccination has resisted the test of 1500 variolous inoculations. In the same

---

by Dr. Adams, that it would be an injustice to the profession not to pointedly recommend his "*Popular View of Vaccine Inoculation*, &c." But while we recommend the whole of this ingenious essay, we cannot refuse to give that sort of publicity to the following fact, which the extensive circulation of the Med. and Phys. Jour. insures. "It is generally thought best to take the fluid before the 8th day; but when this is done *the vesicle from which we take it should be watched till scabbing, for if without other violence than the above puncture the contents should become mattery, or the scab soft, we ought not to depend on the issue of our vaccination.*"

same Presidency, between September, 1802, and the 31st. of August, 1806, there have been successfully vaccinated 607,895 persons.

But little apprehension can now remain that the discovery of Dr. Jenner, pursued with the mild spirit of phylosophy, and explained with truth and candour, will afford the proudest triumph to science, by the annihilation of the small-pox. But while that disease remains we shall, however, be indebted to Dr. Adams for observations that tend to render it more mild than under former inoculations. Dr. Jenner, in his enquiries into the nature of the cow-pox, speaks of a particularly mild sort of small-pox, which spread for some time over Gloucestershire. Of this no other description is given than that it never became confluent; that a given number of cases proved as mild as the inoculated disease; and that the lower class of people so entirely lost all terrors of it, that the usual intercourse was maintained as if no contagion existed. It is suspected, from its universal mildness, that this must have been what nurses call the white sort; the most striking peculiarity of which is, that, contrary to what Sydenham observes, both of the confluent and distinct, the pustules remain white on the face, as well as on the rest of the body, till they begin to scab. In this sort the pustules are never very large, but round and uniform in proportion as the disease is well marked. As they increase, the upper surface extends over the base; and as they dry the scab becomes nearly globular: that is, the whole is distinguishable above the skin, without concealing more of the sphere than what would happen were such a figure actually placed upon the surface. At this time the scab is become of a pale amber, and dries much harder than in the common distinct sort. Its deviations may be defined by the earlier approach of the pustule to yellow, and by the greater softness and inequality of the surface of the scab. By continuing with great caution to inoculate at the Small Pox Hospital, Pancras, from this variety of small-pox, and afterward by selecting those arms which had most the appearance of cow-pox, Dr. Adams at last succeeded in procuring a succession of arms so nearly resembling the vaccine, that a universal suspicion prevailed that one had been substituted for the other*.

In

* This ingenious physician, to whom the profession is much indebted for his faithful observance of the phenomena of a particular class of diseases, has

In the first Number of this Historical Register of the Progress of Medicine, some slight observations were made on the importance of the question respecting the existence of contagion in certain febrile diseases. The year 1807 has furnished additional materials for serious reflection on this weighty point. Two physicians of much eminence have taken opposite sides in the question; and a statement of their opinions, and the facts by which they are supported, will probably give an intelligible view of the subject. Dr. Edward Miller of New York, in a report on the malignant fever which prevailed in that city in the autumn of 1805, has laboured to prove, that *Typhus icteroides* is never disseminated by contagion; while Dr. Gilbert Blane, in a letter to Baron Jacobi Kloest, supports, with equal energy, the contrary opinion.

*Typhus icteroides*, the subject of this diversity of opinion, was first noticed under the name of *Maladie de Siam*, by the Pere Labat, who resided in Martinique about the end of the 17th century. In 1729 or 30, it was epidemic for the first time at Carthagena in South America, where it received the appellation of *vomito prieto*. About the same period, or a few years before, it appeared in the island of Barbadoes. The first notice of it in North America was at Charleston in South Carolina, in 1732; where it again appeared in 1739, 1745, and 1748. It was first known at Philadelphia in 1762; but did not again return till after thirty years, when numbers of French emigrants took refuge there. Since it has been several times epidemic at that place, as well as at New York, and other towns of North America. It was first observed on this side of the Atlantic at Cadiz, in 1764; and again in 1800. In 1803 it appeared at Malaga; and in 1804 it visited the town and fortresss of Gibraltar; spreading the same year to several ports of the Mediterranean, as far as the borders of Valentia; and on the ocean as far as St. Lucar.* The

---

has given, in the work before noticed, very judicious directions for conducting small-pox inoculation, under the following heads:—1st. Season of the year. 2d. State of the person to be inoculated. 3d. Choice of the variolous fluid. 4th. Manner of inserting the fluid. 5th. Treatment of the patient. Those who from opinion or from necessity still inoculate for the small-pox, will be rewarded for the trouble of consulting the work from which the above is extracted, by gaining a clearer view of a disease, which, though wearing out, is yet of serious importance.

* This brief historical statement of the progress of yellow fever in modern times, is collected from documents found in the writings of physicians who

The mortality which marks the progress of this disease is so extensive, that a clear and distinct developement of its cause, has become not only a subject of anxious solicitude, but of serious importance.

Dr. Miller, in his elaborate Address, asserts that a *pernicious vapour, floating in the atmosphere, is its primary and essential cause; and that the disease itself is absolutely and universally non-contagious.* This he endeavours to prove,

1st. From the locality of the disease.

2d. From its appearance and disappearance at particular seasons; and,

3d. From those about the sick being exempt from the disease, when removed from the impure atmosphere.

The noxious miasmata of putrefaction, exhaled by heat, he considers as the material of this *pernicious vapour.* Its peculiarities are,

1st. That it arises from low and moist grounds, overspread with the corrupting offals of animal and vegetable substances, collected in large masses, or where the process of putrefaction is going on to a great extent.

2d. That it is more frequently and copiously produced, and more highly concentrated in warm and tropical countries, than in high latitudes and frozen regions.

3d. That it is generated more in summer, and operates more powerfully in autumn, than in other seasons of the year; and is uniformly more frequent and virulent in sea-ports, along the sea coast, in plains and near rivers, than in the interior high and mountainous districts.

4th. That it is one of the most universal causes of disease in nature; that, however diversified in quantity or virulence by local circumstances, or by varieties of climate, season, or condition of society, its effects, in one degree or another, are nearly co-extensive with the habitable parts of the globe.

Though this noxious exhalation is stated to be the primary who have observed it, at various periods, since the time of Labat. The American physicians would carry a knowledge of it as far back as the time of Hippocrates; and allege, that even a slight inspection of his works will prove that he had observed it under its most malignant and fatal forms. It has been contended that yellow Fever is a modern disease, and utterly unknown to Europe, except when imported from America. To obviate this imputation, the American practitioners cite Hippocrates to prove its existence in the earliest periods of medical knowledge; Baglivi and Lancisi, as evidence of its occurrence at Rome; and Cleghorn, to shew that it is indigenous in the island of Minorca.

mary and essential cause of yellow Fever, yet it is admitted to require, generally, the operation of secondary causes to bring it into action. These are, exposure to heat, fatigue, cold, intemperance, fear, anxiety, &c. And it is acknowledged, that the exciting causes, by suddenly producing the disease, are often so striking, as to lead many entirely to overlook the agency of the atmospheric poison.

It is pointed out by Dr. Miller, in a minute description of the local peculiarities of New York, why this noxious exhalation, or poisonous vapour, has been accumulated there to a quantity and virulence, productive, at various periods, of the *Typhus* icteroides. As it is asserted, that this vapour is a universal cause of disease, in every part of the world, it may not be useless to relate, in Dr. Miller's own words, the circumstances that occasioned its poisonous accumulation at New York.

"The city of New York lies in N. lat. 40, 42, 8; W. long. 74, 9, 45; at the confluence of the river Hudson and Long island sound;* and on the southern and narrow extremity of Manhattan island, which is about fifteen miles in length, and from one to two in breadth. The scite of the city, as it originally stood, was very irregular, being broken into hills and declivities, and indented with small rivulets or creeks, skirted with marsh. Many of the hills are levelled; but the marshy grounds, though covered with houses and pavement, are still low and moist. The city is about twenty-seven miles from the ocean, and is washed on both sides with water of great depth, whose current is very rapid, whose tide ebbs and flows about six feet, and which is nearly as salt as that of the neighbouring sea. On both sides of the city, considerable encroachments have been made on the water by artificial ground, the whole extent of which, may be computed at not less than 132 acres. Of this, 90 acres lie along the East river, and 42 along the Hudson. The portion of it on the East river, forms that part of the city, where malignant fevers have always first become epidemic, and chiefly prevailed. The wharves and docks are constructed of logs and loose stones. All the fresh water used by the inhabitants is procured from wells within the city, and is now become extremely impure. The population of New York may be estimated at about 76,000. The sources of pernicious exhalation

* The East River.

halation in this city, are unhappily very numerous, and difficult to correct. The modes of constructing the wharves and slips, would almost induce the belief that they had been designed for the repositories of filth, and nurseries of disease. The *made ground* on the East river, is pregnant with almost annual pestilence. Originally composed of the most corrupt materials, from its relation to the river, and the condition of the wharves and slips, it must constantly remain moist; from its surface being nearly level, it receives and retains the collected filth washed down from the higher grounds: and beside this, the offensive and putrid matter, which a crowded population must necessarily deposit, and which already underlays a great proportion of this part of the city, incessantly augments the mass of corruption." It can be no matter of surprize, Dr. Miller further observes, that the scorching heat of summer, operating on the complicated pollution of this ground, formed of an aggregate of nuisances, and still the receptacle of numberless others, should exhale poison and death into the atmosphere which stagnates over its surface.

Having pointed out the peculiar atmospheric poison that is said to produce this dreadful epidemic, the circumstances that induce its local accumulation, and some of the laws by which it is governed, we *seem* to have arrived at a determinate and fixed point, in accounting for the source and origin of yellow Fever. This alleged source of the American epidemic, Dr. Miller further supports, by stating under several positions, the reasons for not admitting its propagation by contagion.

After some general remarks on the laws governing contagion, and their operation in Small-Pox, where they are more distinctly ascertained than in any other disease; he proceeds to observe, that the agency of contagion, in the propagation of the malignant American Fever, is rejected, because,

1st. No relation is observed between the source of the pretended contagion, and the spreading of the disease to individuals or families.

2d. The pretended contagion is admitted to produce no effect in the climate of America, except in particular situations, and a particular season of the year, when an impure and noxious atmosphere is said to exist.

3d. It is admitted that the disease does not spread, when the sick are removed from the impure air in which it was contracted.

4th. No

4th. No communication of the disease was ever observed in yellow-fever hospitals, situated at a small distance from the cities to which they belong.

5th. The extinction of the disease by cold weather, is an insuperable objection to the doctrine of its propagation by contagion.

6th. Yellow fever does not prevail in countries where the heat is not sufficient to exhale the miasmata of putrefaction, in the requisite quantity and virulence.

7th. In no instance did those persons who contracted the disease at New York, and died in distant towns and villages, communicate the fever by contagion.

8th. Among the earlier cases of the disease, which were, as usual, most virulent, very striking examples of its non-contagiousness were displayed in some of the most crowded quarters of the city.

9th. The universal exemption of the physicians of New York, amounting to fifty or sixty persons, from the disease, is irreconcilable with the doctrine of contagion.

10th. The failure of every attempt to arrest the progress of the disease, by separating the sick from the well, is also incompatible with the doctrine of contagion.

11th. The inconsistency and contradiction which constantly attend the application of the doctrine of contagion in this disease, make it altogether inadmissible.

If Dr. Miller has not been completely successful in proving that *Typhus icteroides* is the progeny of a *pernicious vapour being the noxious miasmata of putrefaction exhaled by heat*, he very forcibly states the objections to the doctrine of contagion.

The Supreme College of Medicine at Berlin, having offered a Prize for Essays on the nature of the Contagion in Yellow Fever; Dr. Blane, though not as a candidate, addressed some observations to Baron Jacobi, on this obscure subject. The object of the Prussian government was to ascertain the probable danger of this disease making its way into the Prussian territories; and Dr. Blane, by stating some laws by which it is governed, plainly shews the improbability, if not the impossibility, of its spreading to the northern states of Europe.

It is an extremely curious fact in the history of the Plague, that a degree of atmospheric heat below that of the tropical, and above the point of congelation, is essential to its existence. That the cold of winter and the heat of summer are equally inimical to it. Between this fact and a law in *Typhus icteroides*, there is a striking analogy; with with this difference however; the Yellow Fever exists only in tropical heats, and the Plague in a temperature of the atmosphere, between the heat of summer and the cold of winter. This fact in the histoiy of the American epidemic affords a security to the northern parts of Europe, both against its introduction by contagion, and its spontaneous origin from the pernicious vapour of putrid swamps.*

On the subject of this address Dr. Blane has endeavoured to establish two important points.

1st. That the Yellow Fever is contagious.

2. That this contagion is limited by its being a *sine qua non* to its efficiency, that the therm. of Farenheit should be at, or above, 80; and that the effluvia from living human bodies should be in a certain degree of concentration.

The first of these propositions he supports by the recital of a number of facts, authenticated by having come under his own observation, or given on respectable authority. The first of them is the communication of the disease to the Thetis and Hussar frigates, from two French armed ships, taken on the coast of America, in May 1795.

In the month of August 1800, a vessel, on board of which some persons had died of the yellow fever in the passage, arrived at Cadiz. After her arrival, the whole crew, passengers, and pilot, were put on shore, and died of it. The contagion, which spread with such desolating effects, over Cadiz, and some other neighbouring towns, was traced to this source. A French ship from the West Indies brought it to Malaga, in 1803;† and the infection was carried from Malaga to Gibraltar.‡

In 1792 it appears to have been imported to Philadelphia by some French emigrants who took refuge there; and Dr. Lind

* "Different species of infection are regulated by different laws. The small-pox propagates itself in all seasons, climates, and situations. The plague cannot exert its virulence but under a range of heat above the 60th, and below the 80th degree of Farenheit's thermometer. *The yellow fever never spreads but under still higher degrees of heat, and in situations where the air is contaminated by human effluvia.*" Blane.

† After disappearing at Malaga in the winter, it reappeared the summer following, which is the only instance Dr. Blane has met with on record, of its prevailing two years consecutively in a temperate climate.

‡ The identity of this fever has been ascertained at Cadiz, by the description of it in the gazette of Madrid; at Malaga, by the description of Dr. Arejula; at Gibraltar, by the accounts sent from thence by the medical attendants of the army; and at Leghorn, by the description of Dr. Palloni.

Lind mentions, on the authority of a sufferer who survived the fever, that it was carried to that place by the clothes of a person being returned to his family, after he had died of this fever at Barbadoes.

The second proposition is also supported by a relation of facts. There is no instance, either in North America or in Europe, of the *Typhus icteroides* appearing except where the temperature of the atmosphere rose to the 80th degree of Farenheit's T. or of its surviving when the heat declined below that point. When it appeared at Gibraltar in 1804, the autumnal heats were greater than had been known at any former period. It has never been known to prevail, but where the effluvia of the living human body existed in a certain degree of concentration; it has never spread into the country among villages and single houses.

From a full view of the subject, Dr. Blane concludes, that *Typhus icteroides* is *contagious*, because it is known to arise, both in ships and on shore, where men were not exposed to any corrupted exhalations of the soil, nor to pernicious vapours of any kind, except such as proceeded from the contaminated effluvia of the living human body. But that it is limited in its desolating effects, by being confined within certain ranges of atmospheric heat, and requiring, in common with other species of infectious matter, a delicate and casual concurrence of incidents, compounded of internal predisposition and external circumstances, to give it deleterious activity.

The extensive views which Drs. Miller and Blane have taken of this epidemic fever, leave still a doubt of its immediate cause.

It is inconceivable that the permanent source alledged by Dr. Miller, and arising out of fixed localities, as at New York, should so seldom produce the disease. How has it happened that the extensive pabulum of disease, consisting of 90 acres of ground, lying along the East River, "*originally composed of the most corrupt materials, and pregnant with almost annual pestilence,*" so rarely produces Yellow Fever? If this proceeds from a long series of years being deficient in the essential degree of heat, let it be shewn by actual registers of the weather, during such periods. Has it arisen from long calms, combined with the high degree of temperature; let it be ascertained, that heretofore such conjunction of calm and heat had not occurred.

For 30 years Philadelphia was free from this visitation. Can it be ascertained that the natural conjunctions of heat and calm were in the year 1792, precisely what they had been been in 1762; and that in the interval of 30 years, no coincidence of atmospheric influence had arisen to call this pernicious vapour from its putrid resting place?

It appeared at Cadiz twice within an interval of 36 years. Can it be ascertained that in 1764 and in 1800, the heats and calms in the atmosphere of Cadiz were similar; that the exhalations from the earth in those years had the same qualities; that the inhabitants were accumulated in a like degree; that the want of cleanliness in the town was to the same extent; and that in the intervening 36 years no such concurrence of incidents happened? Why has the disease occurred but once at Gibraltar? It is in part answered by the autumn of 1804, being hotter than any other in remembrance. But did the thermometer never stand at 80 and upwards in any other autumn than that of 1804? or were the secondary causes of fear, fatigue, cold, intemperance, &c. more cogent in that year, than in any preceding period? Do the localities of Gibraltar agree with Dr. Miller's first proposition, that "the pernicious exhalation arises from low and moist grounds?"

It seems a natural inquiry, why this pernicious vapour, "the most universal cause of disease in nature," so seldom operates in those countries where *Typhus icteroides* occurs in a most unequivocal shape; and why, from so universal a cause, evident effects are not produced in places affording every collateral circumstance for their generation? These difficulties are all obviated by admitting an imported contagion, at a period when the secondary causes are in full power.

But the statement of Dr. Miller, which proves, that no relation was observed between the source of the contagion and the extension of the disease; that it did not spread but in particular places; that the medical attendants at New York remained uninfected; that persons in the disease did not communicate it when removed into the country, almost forbids the admission of this cause.

Under these opposing theories, each supported by strong facts, each liable to serious objections; the Inquirer after Truth, left in a state of indecision between the Vapour of Dr. Miller and the animal Contagion of Dr. Blane, looks forward to that period when correct registers of the weather shall have fully determined the state of the atmosphere for a series of years, in those places where *Typhus icteroides* occurs, both during the prevalence of that disease, and in its absence. Surely, if Dr. Miller's opinion be just, a proper examination of the atmosphere, aided by the nice discrimidiscriminations of modern chemistry, will detect some varieties in the qualities or impregnations of the air, during a healthy or a sickly season. If Yellow Fever is produced by a *pernicious exhalation or vapour floating in the atmosphere*, may it not be reasonably expected, that some of its qualities may be ascertained; at least, that its actual presence may be demonstrated. It seems of such importance to determine the nature of the atmospheric impregnation, existing as the cause of Yellow Fever, that it would, perhaps, be a mark of wisdom in all governments to establish the most rational means of acquiring this knowledge, and enforce their due fulfilment by laws as rigorous as those of quarantine.*

Fevers, generally, have received a degree of attention during 1807, to which they are entitled, from their magnitude among the morbid affections that constantly attend on human life. In an elaborate Inquiry into the seat and nature of Fevers, as deducible from its phenomena, causes, and consequences, Dr. Henry Clutterbuck argues, that the disease consists in a *topical affection of the Brain, founded in Inflammation.* This hypothesis he supports, by a minute investigation of the symptoms of febrile affections, which are all deducible, the author apprehends, from a disordered state of the parts within the cranium. He has met with a severe, but able and acute animadverter in Dr. Beddoes; who, in Researches, Anatomical and Practical, concerning Fever, as connected with Inflammation, shews that disease as frequently to arise from local affections of other parts as of the brain; and that

* The use of the mineral acid gases is pursued on the continent with an energy that argues a conviction of their efficacy. From experiments in the hospital of *Val-de-Grace*, by *M. Desgenettes*, it appears that the oxymuriatic gas not only checks the propagation of contagious diseases, but contributes to their cure. *M. Pinel* employed it with similar success in the wards of the *Hospital de la Salpetriere*. In Spain the government has encouraged the practice by confering rewards on those who employ it. A machine, invented by *de Morveau*, has been used, it is said, with desired success in disinfecting air; and the experiments that point out the efficacy of this machine, have also determined the prophylactic properties of the mineral acid fumigations, especially as a security from the *contagion of Yellow Fever*. The physician *Cabanellas* shut himself up, with his two sons and forty-eight other persons, in the hospital at Carthagena, in which a great number of persons had perished by Yellow Fever. After forty days residence, with merely the precaution of using mineral acid fumigation, they experienced not the slightest inconvenience. This is, perhaps, as strong a proof of the noncontagious nature of the disease, as of the efficacy of the preventive.

the the opinion of its arising from topical affection of the brain, if it were just, has not the merit of originality, "having been adopted by Rhumalius, at Nuremberg, in 1624; by Dr. Marteau de Granvilliers, in France; by Dr. Wendelstadt of Wetzler, in 1794; and still more recently by Ploucquet, Professor at Tubingen."

Dr. A. Phillips Wilson, in an Essay on the Nature of Fever, attempts to ascertain the principles of Treatment, by an investigation of the proximate cause. A republication of a small Treatise on the cause of Yellow Fever, and the means of preventing it in places not yet infected, by the once celebrated Thomas Paine, deserves notice for a forcible simplicity of style, and clearness of argument. In an Inaugural Dissertation at Edinburgh, *de Typhi Remediis*, by Dr. Baldwin Wake, the beneficial effects of *Gestation* are enforced both from his own experience, and from facts furnished by his friends. The most remarkable cases occurred from the compulsory removal of soldiers from hospitals in the worst weather. In these instances, the benefit was too manifest to escape notice. In the removal of the sick, in the spring of 1794, from the hospital at St. Guislain to Dendermonde, a journey of four days, many instances of relief from the most distressing symptoms were experienced: and in the most severe weather, with frequent falls of snow; more than one hundred patients, labouring under fever, were removed in open carriages, from Embden to Bremen, with the happiest effects. It was observed that delirium, in particular, was abated. Dr. Wake's observations lead him to conclude, that gestation is, 1st. most beneficial in the last stage of fevers; 2nd. that it should be performed in an open carriage, in order that air may be freely admitted, and that the patient may be amused by a succession of new objects; 3rd. that it should be continued for eight or ten hours daily, till the patient begins to recover. The strong facts related by Dr. Wake, should call the attention of the faculty to this means of relieving some of the symptoms of fever, probably by carrying off a portion of the accumulated caloric. There are few practitioners who have not seen fevers suddenly relieved by means not ascertained or not understood, and which have probably been an exposure to cool and fresh air. To patients who have been shut up in the wards of an hospital, or immured in the hold of a ship, steeped in poisonous effluvia, the "breath of heaven fresh blowing," must always be an exhilarating cordial, and often an effectual remedy.

To the Physicians and Surgeons of the Army and Navy, medical science has been indebted for many new facts in the history of diseases; for discoveries that illustrate the principles of physiology; and for manifold improvements in the practice of the art. Diseases of certain occurrence, and, heretofore, of deplorable mortality, have their ravages now so subdued, as never to be formidable, as hardly to excite alarm. The condition of the medical department of the Army has been improved to a certain degree; but in the Navy it has arrived at that point of perfect scientific discipline, as almost to insure health in all climates, and at sea, for any length of time. When the Navy of England is, perhaps, the last barrier between the absolute domination of an insatiable usurper, and the remains of the independence of nations, what does not mankind owe to those persons whose genius and industry have, by securing health to our sailors, enabled the British Fleets to keep the sea for almost an indefinite time.

It will afford the highest exultation to the patriot, the philanthropist, and the philosopher, to know that the medical discipline of the fleets of this country has now reached such absolute perfection, as to make it not uncommon for ships to keep the sea for thirty weeks, and return then to port in high health. A three decked ship, in a cruise off Ushant for twenty-nine weeks, lost not a single man by sickness: and another, the Defiance, maintained a station off L'Orient for eleven months; and when she returned to port was again ready for sea in a week. Chacing squadrons perform voyages to the West Indies, America, and Newfoundland, and return to England fit to go into action. In former wars, a fleet could not make a voyage to the West Indies or America without being laid up in port for many weeks with sickness: and the Grand Fleet, after what would be considered in the present day a very short cruise, was detained at Spithead for a considerable time, having filled Haslar Hospital with its sick, and then being compelled to erect tents on shore to receive many which that spacious building could not contain.

In the history of this improved state of health in the English Fleets, we recognize the profound knowledge, the scientific views, and the patriotic spirit of the Earl St. Vincent. His Lordship, when a private Captain, evinced such attention to the internal economy of his ship, as always inculcated and enforced the only means by which sickness can be prevented. But it was not till he acquired the rank of a flag officer that his system operated generally

 on on the Navy. Old mariners were then astonished to find the health of a Fleet comparatively greater at sea than in harbour. This truth stands recorded in the fact of the Channel Fleet, consisting of twenty-four sail of the line, when under his Lordship's command in 1800, returning to Torbay, and having occasion to send no more than sixteen men to an hospital, after being at sea near seventeen weeks without a day's fresh provision. The orders issued by his Lordship, and which affected this mighty change, evinced, by their rational simplicity, a perfect knowledge of the subject. They went principally to the establishment of sick berths remote from the healthy part of the ship's company; to enforce attention to cleanliness, not by drenching the decks with water, but by dry-rubbing them with sand and stone; to strict ventilation, and the regular airing of beds and bedding; to adapt clothing to the season and climate, particularly enjoining the use of flannel in the winter, as one means of preventing pulmonic affections; and to the securing a plentiful supply of the native vegetable acid.

The author of these pages had felt, jointly with many others, that the higher ranks in society had been inattentive to a department of science, on the due execution of which much of the welfare and happiness of social life depended; and had expressed himself with a warmth only to be justified by a sense of the importance of the subject, and a conviction of the benefits that might arise to the community, by giving that patronage to the art of medicine, which an honest exercise of it deserved. By a tribute to the genius and talents of a person of high consideration, not only as the conqueror of our enemies, but as the preserver of the *health, discipline, and strength* of our Navies, he has, however, made the *amende honorable*; and gratified will he be in the highest degree, if each succeeding year shall enable him to record the well directed benevolence, and the philanthropic views of men, from whom the accidents of birth, fortune, and education, demand an earnest exertion for the protection and encouragement of an *art*, the only object of which is the alleviation of Human Misery.

*Princes Street, Cavendish Square,*

*June,* 1803.

TABLES

